# Effects of social experience on abstract concepts in semantic priming

**DOI:** 10.3389/fpsyg.2022.912176

**Published:** 2022-09-02

**Authors:** Zhao Yao, Yu Chai, Peiying Yang, Rong Zhao, Fei Wang

**Affiliations:** ^1^School of Foreign Studies, Xi’an Jiaotong University, Xi’an, China; ^2^School of Humanities, Xidian University, Xi’an, China

**Keywords:** abstract concepts, social experience, semantic priming, lexical processing, types of concepts

## Abstract

Humans can understand thousands of abstract words, even when they do not have clearly perceivable referents. Recent views highlight an important role of social experience in grounding of abstract concepts and sub-kinds of abstract concepts, but empirical work in this area is still in its early stages. In the present study, a picture-word semantic priming paradigm was employed to investigate the contribution effect of social experience that is provided by real-life pictures to social abstract (SA, e.g., *friendship, betrayal*) concepts and emotional abstract (EA, e.g., *happiness, anger*) concepts. Using a lexical decision task, we examined responses to picture-SA word pairs (Experiment 1) and picture-EA word pairs (Experiment 2) in social/emotional semantically related and unrelated conditions. All pairs shared either positive or negative valence. The results showed quicker responses to positive SA and EA words that were preceded by related vs. unrelated prime pictures. Specifically, positive SA words were facilitated by the corresponding social scene pictures, whereas positive EA words were facilitated by pictures depict the corresponding facial expressions and gestures. However, such facilitatory effect was not observed in negative picture-SA/EA word conditions. This pattern of results suggests that a facilitatory effect of social experience on abstract concepts varies with different sub-kinds of abstract concepts, that seems to be limited to positive SA concepts. Overall, our findings confirm the crucial role of social experience for abstract concepts and further suggest that not all abstract concepts can benefit from social experience, at least in the semantic priming.

## Introduction

Abstract concepts (e.g., *freedom*) are cognitively more complex compared with concrete concepts (e.g., *cat*) because they do not possess a bounded, identifiable object as referent, thus, their perceived content is more variable both within and across individuals. The way in which abstract concepts are acquired and represented has become a topic of intense debate in recent years, especially after the emergence of the embodied approaches to cognition (Yee, 2019). Within this framework, an integrate embodied view, multiple representation theory was proposed, suggesting that abstract concepts could be grounded in sensorimotor systems (e.g., visual and motor information) like concrete concepts, but they would activate to a larger extent linguistic, emotional, and social experience (Borghi et al., 2017, 2019; Pecher, 2018). Previous studies have provided plenty of behavioral and neurophysiological evidence on the role of linguistic experience (Louwerse and Jeuniaux, 2010) and affective experience (Kousta et al., 2011; Vigliocco et al., 2014; Yao et al., 2019) in grounding of abstract concepts. However, the role of social experience in abstract concepts has not received adequate attention (Davis et al., 2020; Fini et al., 2021).

Until recently, the Words As social Tools (WAT) view explicitly emphasizes the importance of social experience for abstract concepts, proposing that social experience is a constitutive part of abstract concepts (Borghi et al., 2019). Previous studies that used a feature listing or property generation task reported that participants were more likely to list communicative acts, social actions, or feelings as properties for abstract concepts than for concrete words (Wiemer-Hastings and Xu, 2005; Recchia and Jones, 2012). Neurophysiological studies also found that abstract concepts could activate brain regions underlying social cognition (e.g., medial prefrontal cortex, superior temporal sulcus; Wilson-Mendenhall et al., 2011; Roversi et al., 2013; Wang and Bi, 2019).

Evidence from different cognition tasks has shown that abstract concepts processing can involve more sociality features (Pecher, 2018). For example, Fini et al. (2021) employed human-avatar motor interaction and concept guessing tasks and found that participants needed more hints from partners in order to guess abstract concepts. Zdrazilova et al. (2018) used a taboo task in which participants were asked to communicate words’ meanings to a partner without using the words *pre se*. By analyzing verbal and gestural data, they found that participants’ speech referenced more people and introspections during communicating the meanings of abstract words.

Moreover, an important advance on the grounding of abstract concepts is the recognition that they are not a unitary whole, but composed of different sub-categories of abstract concepts exist (Ghio et al., 2016; Mkrtychian et al., 2019; Villani et al., 2019). Several studies that used a feature production task (Harpaintner et al., 2018) or performed meta-analyses (Desai et al., 2018) suggested that abstract concepts can be quite different from one another in terms of the features they activate, such as numerical, emotional, moral, and theory of mind. Neurophysiological evidence also showed that specific brain responses were separately engaged for social, emotional, and numerical concepts processing (Moseley et al., 2012; Mellem et al., 2016; Dreyer and Pulvermüller, 2018; Bechtold et al., 2019). In short, there is growing interest for considering the category-specific approach that has been applied to research on concrete concepts (e.g., *animal, fruit, furniture*) to examine of fine-grained abstract categories.

Based on recent work on the role of social experience in the grounding of abstract concepts and their categories, it is uncontroversial that social experience is crucial for understanding and processing of abstract concepts, but it is not clear whether it plays the same role in processing different categories of abstract concepts. Some studies have indicated that abstract concepts could be grounded in different aspects of embodied dimensions (e.g., sensorimotor, social, and affective; Ghio et al., 2016; Borghi et al., 2019; Wang and Bi, 2019; Kiefer and Harpaintner, 2020). For instance, an exploratory analysis by Connell et al. (2018) reported that emotional concepts appear to rely more on inner affective experience than non-emotional concepts. A rating study that performed a cluster analysis revealed four categories of abstract concepts, including philosophical emotional, social, and physical concepts. Among them, philosophical concepts were more abstract than the others; physical concepts were more concrete and more linked to interactions with external environment; by comparison, emotional concepts were more characterized by inner grounding, and social concepts relied both on inner and external grounding (Villani et al., 2019). These studies imply that although emotional and social concepts are associated only with inner affective information, but social concepts are grounded by both inner affective and social information.

Given all that, the aim of the present study is to examine whether social experience plays a specific role in the grounding of Social Abstract (SA) concepts, relative to Emotional Abstract (EA) concepts. The core question is whether social experience could be a main embodied dimension for characterizing SA concepts and effectively distinguishing them from other categories of abstract concepts. In the current study, SA concepts refer to general social knowledge that emerges from interpersonal interactions (e.g., *betrayal, duty, loyalty*; Bolognesi and Steen, 2018; Wang and Bi, 2019) and generally have an affective connotation (Giffard and Pratique, 2015). EA concepts refer to basic emotional experience that can directly label individual’s internal affective states (e.g., *happiness, anger, sad*; Kazanas and Altarriba, J., 2015). For example, the SA word “*friendship*” contains a social knowledge of *“a closing and lasting relation between you and a person you like”* and a positively affective experience such as “*happiness*.” In contrast, the EA word “*sad*” only conveys a negative feeling such as “*unhappiness.*”

Although SA and EA concepts lack clear and perceivable referents, they still evoke specific social scenes, episodes, or affective states and in turn are represented in terms of message from pictures depicting people, places, objects, and an individual’s internal state (Barsalou et al., 2008; Wilson-Mendenhall et al., 2011). For instance, the social scene of *two boys with a smile have a snow fight in the park* might help us to understand the meaning of “*childhood.*” By contrast, the meaning of the EA word “*cheerfulness*” could be obtained from *boys’ smiling face and body posture*. Concrete elements, such as *boys, snow fight, smiling faces, and park*, construct a particular social scene that could be experienced first-hand or could be heard or seen and thus effectively represent meanings of abstract concepts. Importantly, these concrete elements of pictures are usually perceived as a whole (Wilson-Mendenhall et al., 2011; McRae et al., 2018; Pecher, 2018). From this perspective, it is believable that pictures of social scenes, facial displays or body gestures automatically trigger relevant concept representation.

In the present study, we employed a “picture-word” semantic priming paradigm in a lexical decision task to compare the contributions of social experience to SA concepts and EA concepts. Semantic priming is a typical paradigm used to examine mental representations of word meanings and their relationships (Meyer and Schvaneveldt, 1971). Semantic priming effect refers to the faster and more accurate response to a target (e.g., *love*) when it is preceded by a semantically related prime (e.g., *marry*), relative to an unrelated prime (e.g., *news*). This effect is usually explained by an automatic spread of activation through semantic memory (Neely, 1991). Because pictures, similar to words, have a direct and functional connection to semantic system, with a similar activation like words in semantic memory (Glaser, 1992; Herring et al., 2013), and thus this paradigm could provide a means of measuring semantic relationship of picture-word pairs.

We conducted two experiments to examine responses to picture-SA word pairs (Experiment 1) and picture-EA word pairs (Experiment 2) in semantically related and unrelated conditions, in which all pairs shared either positive or negative valence. We considered stimuli valence is due to the fact that, in the literature on semantic priming, the difference in priming effects has been found between positive and negative primes (Rossell and Nobre, 2004). Therefore, four types of prime pictures were used, two of them were Social Scene (SS) pictures with either positive or negative valence, describing people’s social interactions in a real-life situation, and the other two were positive or negative Emotional Expression (EE) pictures, describing a person’s facial expression and gesture but without social interaction and situation. Accordingly, SA and EA target words were, respectively, presented under four experimental conditions in Experiment 1 and 2, including social-semantic primes (i.e., SS pictures) and/or non-social semantic primes (i.e., EE pictures), and all prime-target pairs shared either positive or negative valence.

We hypothesized that in Experiment 1, if social experience is a more significant factor for SA concepts than for EA concepts, then SA target words would be more readily facilitated by social-semantic related SS pictures, compared to social-semantic unrelated EE pictures. Conversely, if EA concepts are more detached form social experience or more dependent on inner affective states than SA concepts, then in Experiment 2, EA target words would be more readily facilitated by emotional-semantic related EE pictures, relative to social-semantic related SS pictures. That is, in the lexical priming-decision task, the effect of social experience provided by a real-life situational picture on the processing of SA and EA words might be an opposite. Meanwhile, the effect of social experience on SA and EA words might be modulated by their valence.

## Norming study

We began with a norming study to generate stimuli for the subsequent experiments. A set of positive and negative picture-word pairs were created, consisting of social-semantic related pairs and emotional-semantic related pairs. This stage included four steps: (1) select pictures in term of the definitions of SS and EE pictures; (2) name each picture by reference to the method used in McRae et al. (2018); (3) rate semantic relationship between picture and its name; and (4) match affective and/or lexical variables of pictures and words.

### Selected SS and EE pictures according to their definitions

**Figure 1:**
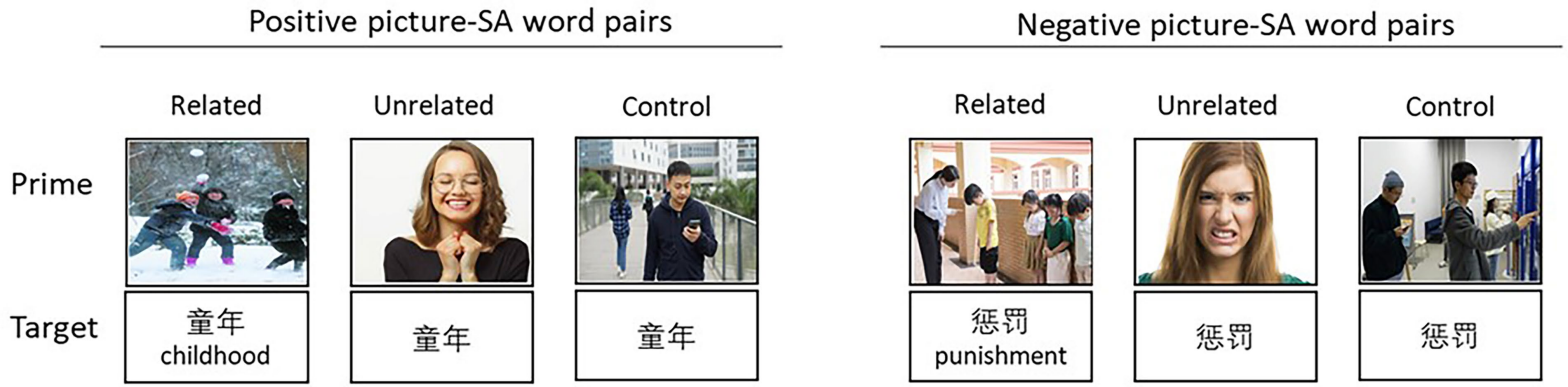
Examples of a social scene picture (left) and an emotional expression picture (right).

We referred to the social–emotional sentences in Mellem et al. (2016) and initially collected 94 SS pictures and 96 EE pictures (half positive and half negative) from International Affective Picture System (IAPS; Lang et al., 2005), the Chinese Affective Picture System (CAPS, Bai et al., 2005) and public photos from the Internet. The selection of pictures was guided by the standard that a picture has a positive or negative affective meaning, and without conspicuous letters. We believed that these pictures convey the meaning of the corresponding words and can elicit consistent responses among participants. Two examples are shown in Figure 1.

### A picture-naming task

#### Participants

Forty undergraduate students (26 males; mean age = 22.4, *SD* = 2.3) were recruited from Xidian University and were asked to provide 2–5 words related to each picture by an online questionnaire survey (SO JUMP, https://www.wjx.cn/). Each participant received 50 RMB for their participation.

#### Procedure and result

The procedure borrowed the method that was used in McRae et al. (2018)’s norming study. Participants were instructed to avoid naming people and objects, but rather to provide words summarizing the whole situation, or the affective states, or thoughts of the people in the picture. Appendix A presents the instructions. The task was self-paced, with all participants completing the task in 3 days.

According to a weighted score of each picture, we got the most appropriate name for each picture. The weighted score was calculated as follows: Rank Sum Score = 5a + 4b + 3c + 2d + e, where a, b, c, d, and e refer to the number of participants who provided that response in ranks 1, 2, 3, 4, and 5, respectively (McRae et al., 2018). The maximum possible rank sum score was 200 (40 participants times 5 if all participants provided the same word as their first response). According to the rank sum score of each picture, we obtained 174 words (16 pictures were given up due to the rank scores below 69). The scores varied between 70 and 162, with a mean of 92. We found in the 174 words there existed general consistency in terms of word-picture semantic relationship: 85 words were elicited from SS pictures, and 89 words from EE pictures.

To insure a reliable social or emotional semantic relationship of a picture and its corresponding name, we additionally conducted a rating of semantic correlation of the 174 picture-word pairs.

### The rating of social or emotional semantic correlation between each picture and its name

#### Participants

Sixty native Chinese speakers (12 males, mean age = 18.4, *SD* = 1.3) were divided randomly and equally into two groups. This rating study was conducted in a public psychology course of Xian Jiaotong University, and all participants read and signed consent to the study and received course credits for their participation.

#### Materials and procedure

One hundred ninety-four picture-word pairs were randomly divided into two parts (97 pairs in each), consisting of 174 pairs chosen from the previous step, and 20 unrelated pairs (without semantic and emotional association) used as filler stimuli to offset the fact that all picture-word pairs were related. The prime pictures in fillers were neutral pictures that were selected from CAPS (Bai et al., 2005).

Two group participants were asked to rate social or emotional semantic relationship of each picture-word pair on the Likert scale ranging from 1 to 7, with 1 indicating *extremely unrelated*, and 7 indicating *extremely related*. All participants completed the task within 15 min.

#### Results

Three participants were removed from the analysis due to a poor performance to filler stimuli (eight out of ten were wrong). We calculated the mean scores and standard deviations for each picture-word pair on semantic relationship in SPSS 26.0 and decided to consider picture-word pairs with a score of 6.13 or higher as the semantic related pairs. As a result, we chosen 72 SS picture-SA word pairs and 68 EE picture-EA word pairs.

In the next step, we collected subjective ratings for pictures and words on several important affective and/or lexical variables that were known to affect behavioural responses.

### The ratings of affective and/or lexical variables for pictures and words

#### Participants

Fifty-two native Chinese speakers were recruited from Xian Jiaotong University, ranging in age from 18 to 25 years (29 males, mean age ± *SD* = 23.4 ± 1.3). They were randomly and equally assigned to complete either affective variables ratings for pictures or affective and lexical variables ratings for words. They received monetary compensation in the end for participation.

#### Materials and procedure

One group completed valence, arousal, abstractness, familiarity, and referent ratings for words on Likert scales ranging from 1 to 9 (1 meant extremely negative/calm/abstract/unfamiliar/label individual inner feelings, 9 meant extremely positive/arousing/concrete/familiar/derived from interpersonal interaction). The instructions for valence, arousal, abstractness, and familiarity referred to our prior work (Yao et al., 2017). The instruction for referent referred to the definitions of SA and EA concepts. Likewise, valence and arousal ratings for pictures were assessed by the other group. Appendix B presents the English translations of the instructions.

The rating task was implemented by the online questionnaire survey (SO JUMP) and was self-paced, completed in 1–3 sessions within 2 days.

#### Results

All participants completed the ratings tasks, and thus no participant’s responses were removed. We calculated mean valence and arousal scores of each picture, as well as mean scores of each word on valence, arousal, abstractness, familiarity, and referent in SPSS 26.0.

Picture and its name (i.e., target word) that were presented to participants in the formal experiments were selected according to several criteria that were contrasted with one-way analysis of variance (ANOVA; see Table 1) and *post hoc* analyses with the Bonferroni correction (*α* < 0.05): positive and negative SS/EE pictures were matched on arousal yet differed in valence. Positive and negative SA/EA target words were matched on arousal, abstractness, and familiarity, but differed in valence and referent. Descriptive statistics for selected pictures and words are summarized in Table 1.

**Table 1:** Descriptive statistics for selected pictures and words samples.

Variables			Valence	Arousal	Abstractness	Familiarity	Referent
Stimuli type							
**Picture**	Positive	SS	6.73 ± 0.44	6.20 ± 0.45			
		EE	6.61 ± 0.42	6.17 ± 0.34			
	Negative	SS	2.99 ± 0.66	6.29 ± 0.47			
		EE	3.05 ± 0.61	6.24 ± 0.33			
One-way ANOVA each factor			*F*_3,92_ = 358.5, *p* < 0.001	*F*_3,92_ = 0.36, *p* = 0.78, n.s.			
**Word**	Positive	SA	6.33 ± 0.46	6.09 ± 0.37	2.28 ± 0.52	6.26 ± 0.50	6.52 ± 0.65
		EA	6.44 ± 0.35	5.92 ± 0.51	2.43 ± 0.63	6.33 ± 0.42	2.14 ± 0.51
	Negative	SA	2.71 ± 0.76	6.02 ± 0.56	2.53 ± 0.60	6.02 ± 0.60	6.94 ± 0.49
		EA	2.87 ± 0.88	6.11 ± 0.54	2.48 ± 0.52	6.26 ± 0.63	1.87 ± 0.42
One-way ANOVA each factor			*F*_3,92_ = 246.1, *p* < 0.001	*F*_3,92_ = 0.68, *p* = 0.57, n.s.	*F*_3,92_ = 0.86, *p* = 0.47, n.s.	*F*_3,92_ = 1.44, *p* = 0.24, n.s.	*F*_3,92_ = 647.0, *p* < 0.001

Means of Valence (1, Negative to 9, Positive), Arousal (1, Calming to 9, Arousing), Abstractness (1, Abstract to 9, Concrete), Familiarity (1, Unfamiliar to 9, Familiarity), and Referent (1, Inner feelings to 9, Human interaction). n.s., nonsignificant; ANOVA, analysis of variance; SS, Social Scene pictures; EE, Emotional Expression pictures; SA, Social Abstract words; EA, Emotional Abstract words.

As a result, the 48 social-semantic related pairs (24 positive and 24 negative SS picture-SA word pairs) and 48 emotional-semantic related pairs (24 positive and 24 negative EE picture-EA word pairs) were created. Part of picture-word pairs is presented in Appendix C.

## Experiment 1: Effect of social experience on the recognition of SA concepts

In Experiment 1, we examined whether social experience that is provided by positive or negative SS pictures facilitates the responses to social-semantic related SA words in semantic priming. It was hypothesized that a significant semantic priming effect could be observed, with a quicker response to SA words that were preceded by SS vs. EE pictures (emotional-semantic related). Moreover, the social semantic priming effect of positive pairs might be different from negative pairs.

### Methods

#### Participants

Thirty-four native Chinese speakers (18 males; 18–24 years old, mean age ± *SD* = 19.8 ± 2.1) were all right-handed (Oldfield, 1971) with normal or corrected-to-normal vision. None of them had history of neurological or psychiatric disorders. Each participant signed a written informed consent before the experiment and received monetary compensation for their participation. The study was approved by the local Ethics Committee of Xian Jiaotong University.

#### Materials

**Figure 2:**
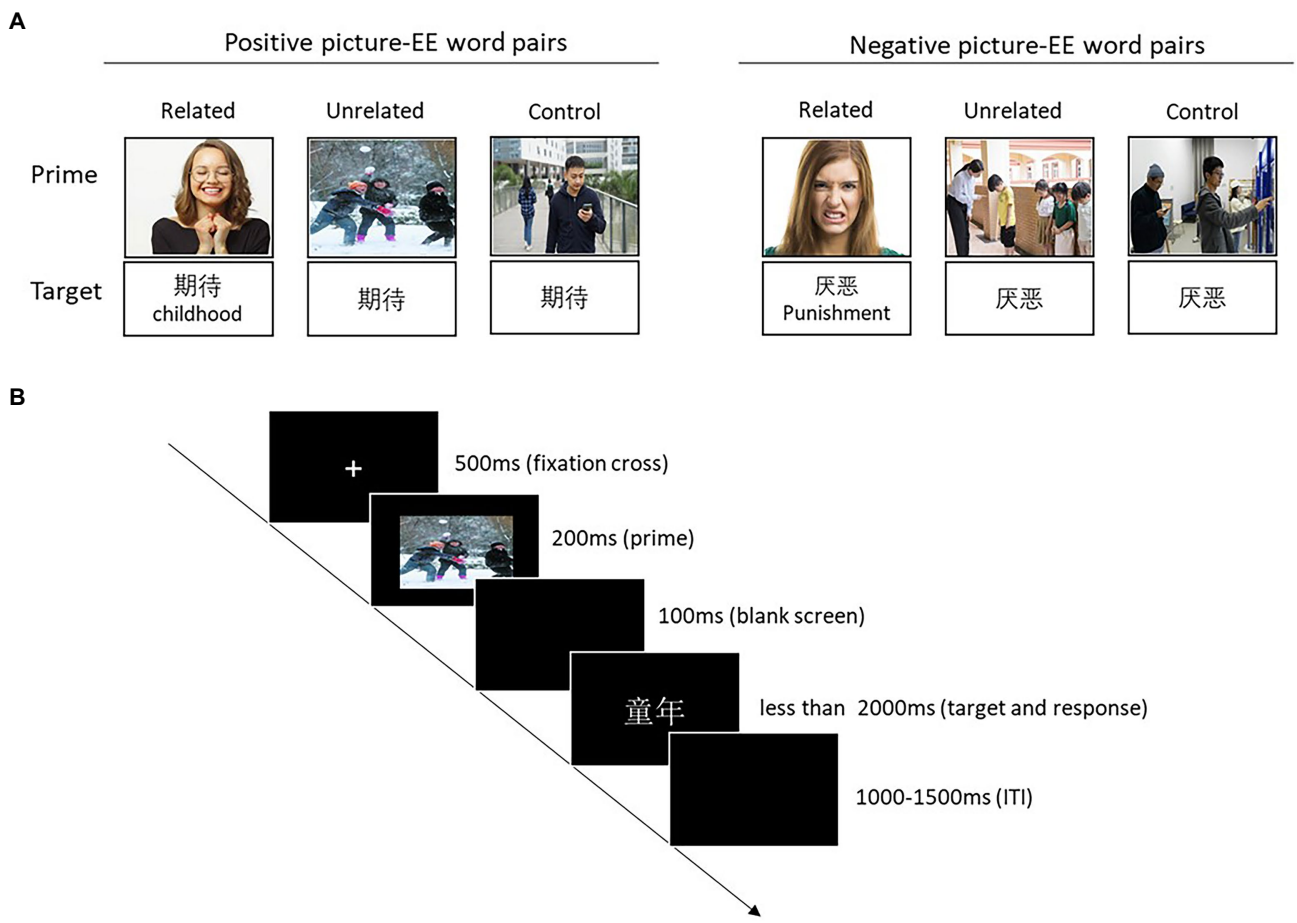
**(A)** Six experimental conditions of Experiment 1. **(B)** Six experimental conditions of Experiment 2.

Six experimental conditions were created according to target valence (positive, negative) and social-semantic relatedness (social related, social unrelated, control) of picture-SA word pairs, with 24 items in each condition (see Figure 2 for examples). Two conditions featured either positive or negative social semantic association between SS pictures and SA words, which were selected from the norming study. No social-semantic association was included in the other two conditions, in which SA words were preceded by EE pictures. All pairs shared either positive or negative valence but without any social semantic association. The remaining two conditions with neutral pictures as primes were used as control conditions, in which positive and negative SA words were preceded by neutral pictures. The 24 neutral pictures were selected from ISIEA database (the image database of social inclusion/exclusion in Asian young adults. Zheng et al., 2021) and described people in social situations that did not involve interpersonal interaction and affective meanings. The neutral pictures significantly differed in valence (*F*_2,117_ = 649.7, *p* < 0.001; 5.06 ± 0.24) and arousal (*F*_2,117_ = 299.0, *p* < 0.001; 4.21 ± 0.14) from positive (valence = 6.67 ± 0.43; arousal = 6.19 ± 0.39) and negative (valence = 3.02 ± 0.63; arousal = 6.26 ± 0.40) pictures.

The relatedness of picture-word pairs in social semantically unrelated and control conditions were rated by another sample of 18 participants using the 7-point scale (a higher score indicates a higher level of social-semantic association). The means and *SDs* of semantic relatedness ratings for all pairs are presented in Table 2.

**Table 2:** The rating scores of semantic relationships between prime pictures and SA words.

	SA words paired with different primes	Semantic relationship
Related	positive SS pictures	6.28 ± 0.98
	negative SS pictures	6.19 ± 1.07
Unrelated	positive EE pictures	2.11 ± 1.68
	negative EE pictures	1.94 ± 0.87
Control	neutral pictures-positive SA words	2.01 ± 1.05
	neutral pictures-negative SA words	1.92 ± 1.25

SS pictures, Social Scene pictures; EE pictures, Emotional Expression pictures; SA words, Social Abstract words; EA words, Emotional Abstract words.

In addition, we used 144 picture-pseudoword pairs. The pseudowords, all pronounceable, were generated by altering one random character within different real words. In short, there were 264 experimental pairs, consisting of 96 social-semantic related or social-semantic unrelated picture-SA word pairs that had either positive or negative valence (24 pairs in four conditions), 24 neutral picture-SA word pairs, and 144 picture-pseudoword pairs.

#### Task and procedure

Participants performed the lexical decision task in separate sound-proof booths. They were told that a picture would be briefly presented on the screen and be immediately followed by a word. They were indicated to respond as quickly and accurately as possible whether the word was a real word or a pseudoword by pressing the “Z” and “M” keys on the keyboard (assignment of the two keys to response categories was counterbalanced across participants).

All 288 trials (24 neutral picture-SA word pairs were repeated one time) were shown in four blocks of 72 trials each. Two of the blocks had positive SA words as targets, with each containing 12 social-semantic related, 12 social-semantic unrelated, 12 neutral, and 36 pseudoword pairs. The other two blocks had negative SA words as targets, and each block contained the same proportion of pairs as positive SA blocks. The order of pair presentation in each block and the order of the blocks was randomized for each participant.

**Figure 3:**
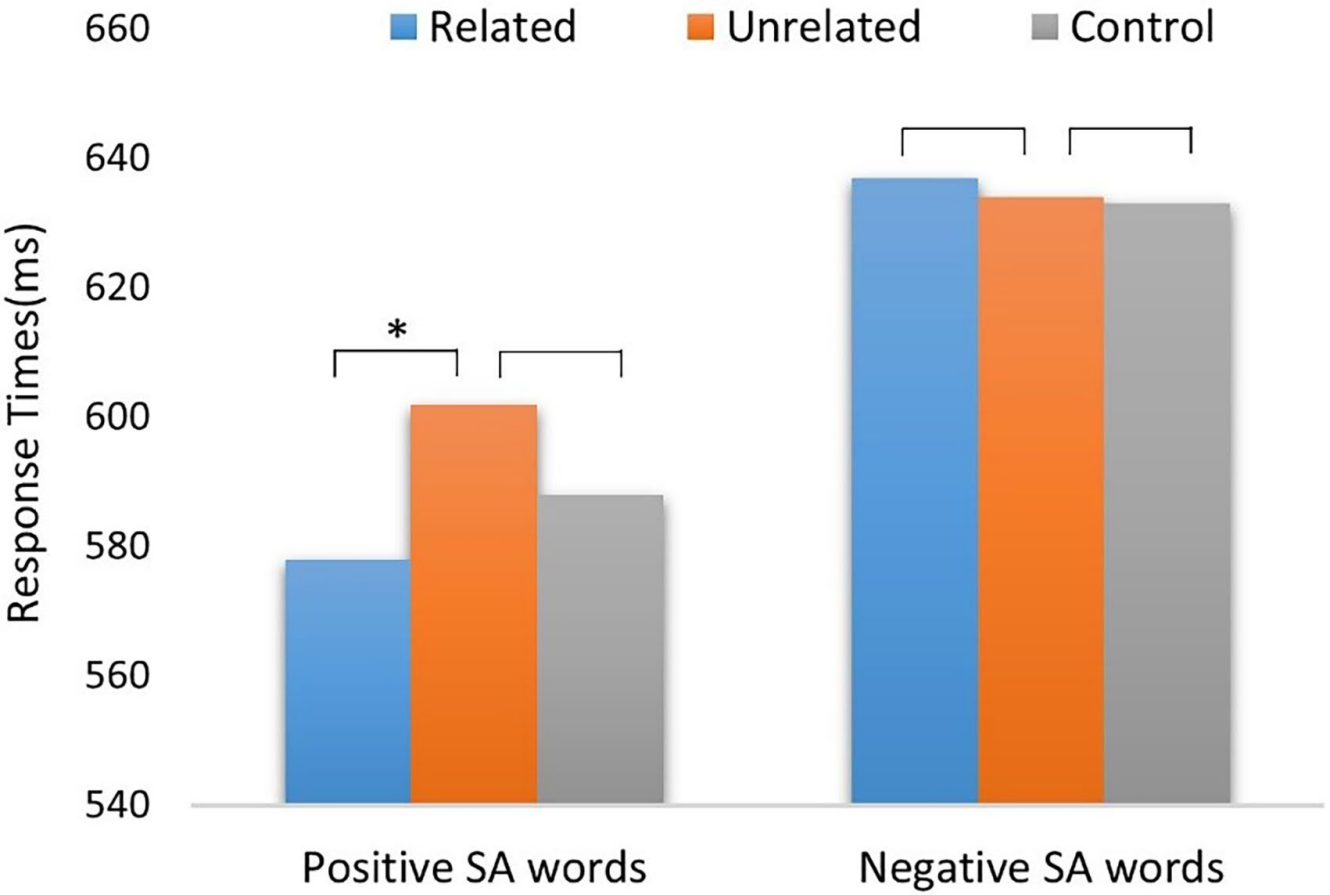
Trial procedure for Experiment 1. “童(tong2)年(nian2) means “childhood.”

Stimuli (prime pictures: 400 × 245 pixels; target words: Song typeface, size: 36) and instructions were presented in white letters over black background on a 21-in. monitor. Each trial started with the presentation of a fixation cross for 500 ms, followed by a prime picture for 200 ms. After the prime, a blank screen was shown for 100 ms before the target was presented until the participant responded or 2,000 ms elapsed. The inter-trial interval was 1,000–1,500 ms (see Figure 3). Prior to the experiment trials, each participant performed 12 practice trials (these trials did not appear in the formal experiment) to prove that they had completely understood the procedure and correct key presses. All experiments were programmed using E-Prime 3.0 (Psychology Software Tools Inc., Sharpsburg, PA).

### Results

One participant with an overall accuracy below 50% was removed from the analyses. In the remaining 33 participants, overall accuracy was high (98.8%) and did not differ between experimental conditions (range: 97.5–99.6%). Therefore, consequent analysis concentrated on response times (RTs). We excluded from the analyses mean RTs above or below 2.5 standard deviations from the mean, and only analyzed RTs for correct responses to target stimuli.

**Figure 4:**
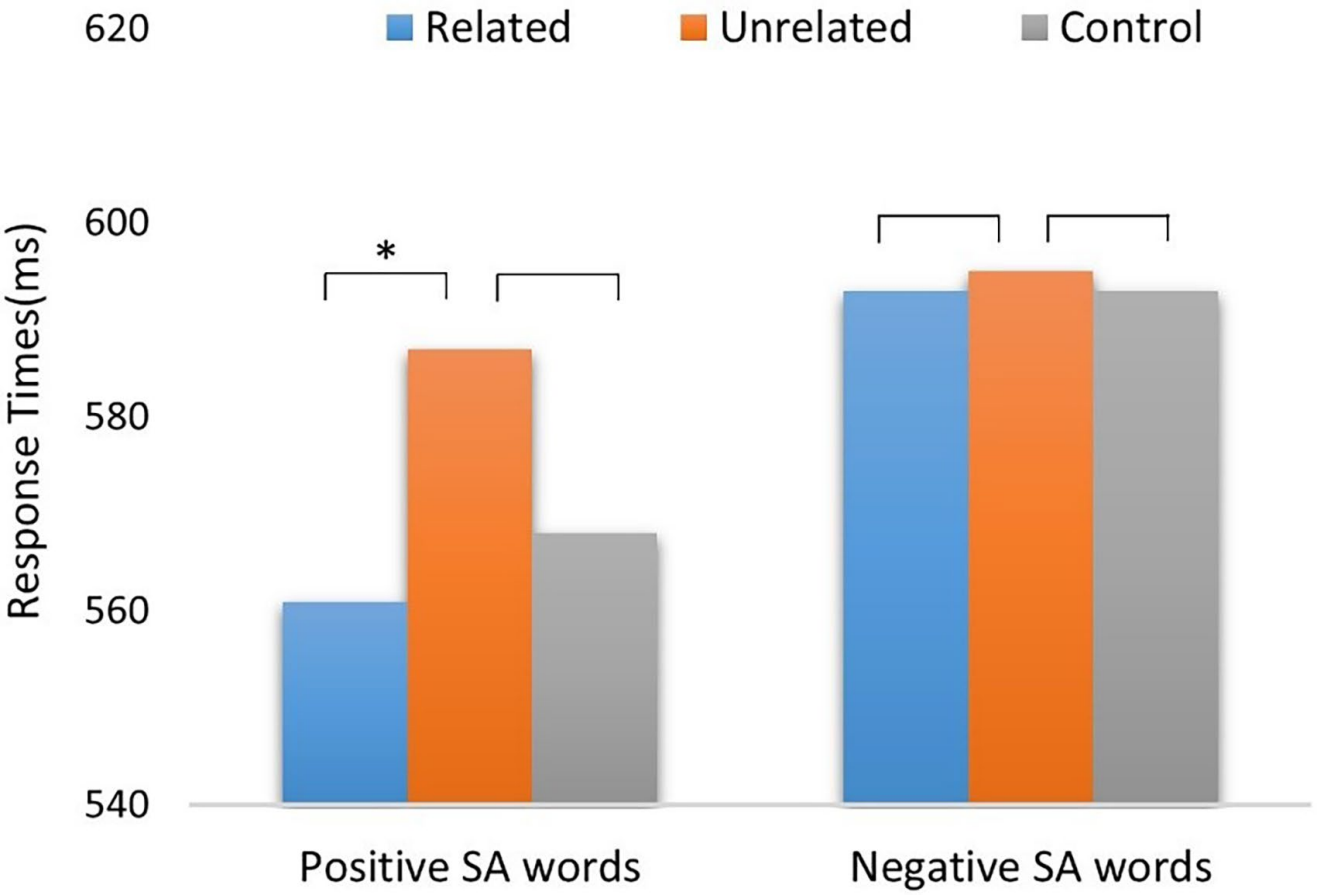
Response times of positive/negative SA words in social-semantic related, unrelated, and control conditions. SA words = Social Abstract words.

A repeated-measures ANOVA was run on RTs in the six prime-target conditions: 2 (target valence: positive vs. negative) × 3 (social-semantic relationship: related vs. unrelated vs. control). The results revealed a significant main effect of target valence (*F*_1,32_ = 34.5, *p* = 0.001, $\eta_{p}^{2}$ = 0.52), with slower responses to negative SA words (634.2 ± 13.3 ms) compared to positive SA words (589.1 ± 13.1 ms). A main effect of social-semantic relationship of picture-word pairs was significant (*F*_2,64_ = 3.72, *p* = 0.03, $\eta_{p}^{2}$ = 0.80), with responses to social-semantic unrelated pairs (617.7 ± 12.9 ms) being slower than to social-semantic related (607.1 ± 12.2 ms) and control pairs (610.1 ± 13.5 ms). A significant interaction was found between target valence and social-semantic relationship (*F*_2,64_ = 3.95, *p* = 0.03, $\eta_{p}^{2}$ = 0.11; Figure 4). The simple-effect analysis showed that responses to positive SA words were significantly faster in semantically related (577.8 ± 12.2 ms) condition than in unrelated (601.9 ± 14.0 ms) and control (587.7 ± 14.4 ms) conditions (*F*_2,64_ = 7.92, *p* < 0.001). However, for negative SA words, no significant differences between related (636.5 ± 13.8 ms), unrelated (633.5 ± 13.3 ms), and control (632.5 ± 14.4 ms) conditions were observed (*F*_2,64_ = 0.2, *p* = 0.82).

### Discussion

In Experiment 1, positive SA words were facilitated by the corresponding positive SS pictures compared with positive EE pictures, showing a significant social-semantic priming effect. However, for negative SS picture-SA words, no significant difference in RTs was found between related and unrelated conditions. The pattern of results suggests that positive SA words could more readily benefit from social experiential information of prime pictures to strengthen the semantic association between them. A possible reason for this result is that most people usually live in a normal social environment, in which people tend to watch, hear, and experience a healthy and positive social interaction. Similar positive bias during processing of words was also observed in many previous studies (Kanske and Kotz, 2007; Hinojosa and Me, 2010; Bayer et al., 2012), which was explained by that the human brain is more reactive to the valence of positive relative to negative words (Yang et al., 2013).

## Experiment 2: Effect of social experience on the recognition of EA words

In Experiment 2, EA words with positive or negative were used as targets, which were preceded by affectively consistent EE or SS prime pictures. We expected that, if EA words are more detached form social experience than SA concepts, then EA target words would not be facilitated by the SS pictures instead of the corresponding EE pictures, showing a different pattern from SA words.

### Methods

#### Participants

Thirty-nine university students (25 males; 17–23 years old, mean age ± *SD* = 22.3 ± 2.4) participated in Experiment 2 and received financial compensation for participation (see Experiment 1 for further details). They all gave a written informed consent prior to the experiment.

#### Materials

Similar to the proportion of Materials in Experiment 1, there were also 264 experimental pairs, with the exception of using EA words as targets (see Figure 2B for examples). Specifically, the stimulus set included 48 emotional-semantic related EE picture-EA word pairs (24 positive, 24 negative), 48 emotional-semantic unrelated SS picture-EA word pairs (24 positive, 24 negative), 24 neutral picture-EA word pairs, and 144 picture-pseudoword pairs. The semantic relatedness of all picture-EA word pairs was also rated using the 7-point scale, with the same sample as in Experiment 1. The rating scores are shown in Table 3.

**Table 3:** The rating scores of semantic relationships between prime pictures and EA words.

	EA words paired with different primes	Semantic relationship
Related	positive EE pictures	6.41 ± 1.36
	negative EE pictures	6.22 ± 1.17
Unrelated	positive SS pictures	2.18 ± 1.58
	negative SS pictures	2.11 ± 1.07
Baseline	neutral pictures-positive EA words	1.99 ± 1.10
	neutral pictures-negative EA words	2.02 ± 0.91

EE pictures, Emotional Expression pictures; SS pictures, Social Scene pictures; EA words; Emotional Abstract words; SA words, Social Abstract words.

#### Task and procedure

The experimental task and procedure were the same as in Experiment 1.

### Results

We excluded from the analyses mean response times (RTs) above or below 2.5 standard deviations from the mean, and only analyzed RTs for correct responses to target stimuli, because the accuracy for each trial in all conditions was high (98.2%, range: 97.2–99.4%) and did not differ across conditions.

**Figure 5:**
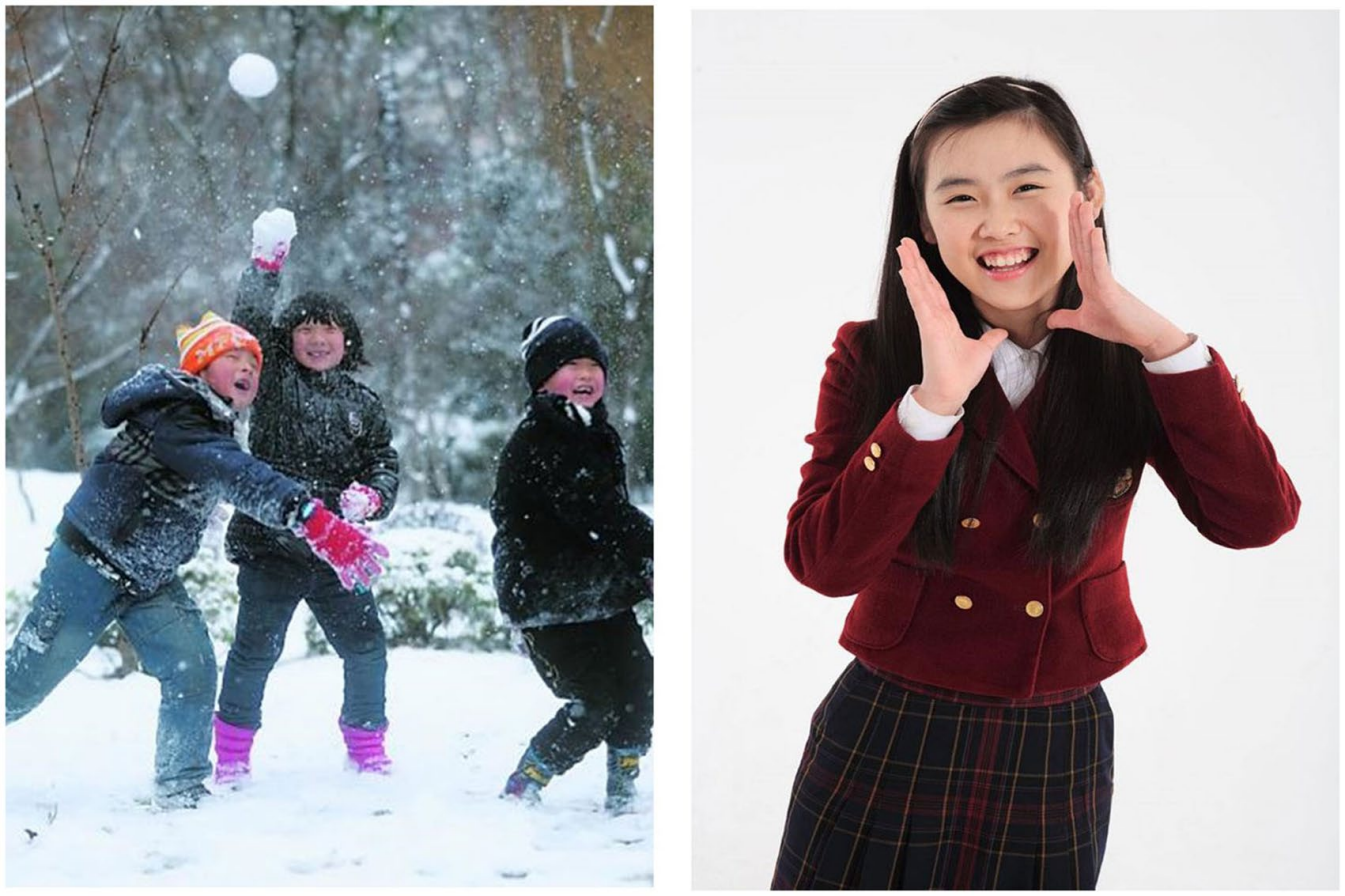
Response times of positive/negative EA target words in semantically related, unrelated, and control conditions. EA words = Emotional Abstract words.

A repeated-measure AVOVA on RTs in six experimental conditions and revealed a significant main effect of target valence (*F*_1,38_ = 17.7, *p* < 0.001, $\eta_{p}^{2}$ = 0.32), with longer RTs in negative pairs (593.6 ± 11.0 ms) than in positive pairs (571.8 ± 10.6 ms). A main effect of emotional-semantic relationship was significant (*F*_2,76_ = 6.52, *p* = 0.002, $\eta_{p}^{2}$ = 0.15): responses to related (577.2 ± 10.8 ms) and control conditions (580.1 ± 10.6 ms) were faster than responses to unrelated conditions (590.8 ± 10.8 ms). A significant interaction between target valence and semantic relationship was found (*F*_2,76_ = 4.11, *p* = 0.02, $\eta_{p}^{2}$ = 0.1; Figure 5). The simple-effect analysis showed that RTs to positive EA words were significantly longer in semantically unrelated (586.5 ± 11.4 ms) conditions compared with related (561.4 ± 10.8 ms) and control (567.5 ± 10.8 ms) conditions (*F*_2,76_ = 12.45, *p* < 0.001), but no significant differences in RTs to negative EA words whether in related (593.0 ± 11.6 ms), unrelated (595.1 ± 11.5 ms), and control (592.8 ± 11.7 ms) conditions (*F*_2,76_ = 0.09, *p* = 0.92).

### Discussion

The results of Experiment 2 shown that only positive EA words were facilitated by emotional-semantic related positive EE pictures relative to positive SS pictures. That is, a significant emotional-semantic priming effect was found for positive EA words, but not for negative EA words. We inferred that positive EA words describing inner positive feelings might more readily benefit from our facial displays and body gestures, thus evoking a tighter emotional-semantic association between positive EE pictures and EA words. By comparison, social experiential knowledge that underlies positive SS pictures seems not to offer an additional resource to accelerate the recognition of positive EA words.

Similar to negative SA words in Experiment 1, negative EA words were not influenced, whether they were preceded by negative EE or SS pictures. This result is consistent with previous studies (Rossell and Nobre, 2004; Sass et al., 2012), suggesting that primes’ negative information inhibits the spread of activation between related concepts, so that target words cannot extract related emotional-semantic content from pictures to build a closer semantic relatedness, thus showing a null effect for negative primes.

## General discussion

In the present study, a picture-word semantic priming paradigm was designed to explore the distinct role of social experience in the grounding of SA and EA concepts. The SA and EA words that shared similar affective and lexical variables were used as targets in Experiments 1 and 2, respectively. The results of the two experiments shown that semantic priming effects were observed for both positive picture-SA word pairs and positive picture-EA word pairs, with quicker responses to semantically related pairs than to semantically unrelated pairs. Note that positive SA words were facilitated by the corresponding SS pictures, whereas positive EA words were facilitated by the corresponding EE pictures instead of SS pictures. This pattern of results suggests that positive social experience from real-life scenes (i.e., SS pictures) facilitates the recognition of related SA words, but not of positive EA words. Moreover, such facilitation was not observed in negative picture-SA/EA word conditions. Overall, these findings confirm the WAT view, emphasizing a crucial role of social experience for abstract concepts, and further reveal that social experience could be an embodied dimension for specifically characterizing SA concepts and distinguishing SA concepts from other types of abstract concepts, such as EA concepts, at least in the positive semantic priming context.

Our experiments show significant semantic priming effects in positive picture-SA/EA words, which are consistent with other studies exploring automatic semantic priming in a lexical decision task (e.g., Rossell and Nobre, 2004; Sass et al., 2012). Such priming effects can be explained by spreading activation within semantic networks. According to spreading activation theory (Neely, 1991), activation is considered to spread from a prime to a target if the two share a closer semantic association in semantic memory, thereby influencing decisions to targets. Thus, quicker responses to positive SA words in related vs. unrelated conditions can be explained by the fact that positive SA words more readily benefit from social experience conveyed by the related SS pictures, and thus strengthen the semantic association between the two. By comparison, positive EA words are more easily accelerated by facial expressions and body gestures that are provided by the corresponding EE pictures. In other words, social experience that is provided by positive SA pictures did not facilitate the recognition of positive EA words. Such findings are in accordance with our hypothesis, showing that sub-kinds of abstract concepts may not equally benefit from social experience, at least in positive semantic priming context.

Moreover, our findings confirm the WAT, which emphasizes the relationship between social experience and abstract concepts (Borghi et al., 2017, 2019; Pecher, 2018; Davis et al., 2020), and also support recent empirical works reporting a facilitating role of sociality in the processing of abstract concepts (Mellem et al., 2016; McRae et al., 2018; Zdrazilova et al., 2018; Fini et al., 2021). For example, McRae et al. (2018) reported that pictures depicting real-world social scenes (e.g., *two girls sharing a corn cob*) could facilitate the processing of related abstract words (e.g., *friendship*) in a lexical decision task, and vice versa. In our study, positive SA and EA words shared similar affective and lexical variables in a parallel semantic priming context, and thus the difference between the two in the recognition performance could be ascribed to the fact that they are grounded in varied degrees of social experience. Specifically, positive SA words are characterized by more social features compared to positive EA words. This finding provides empirical evidence to show that abstract concepts may be quite different from one another (Connell et al., 2018; Harpaintner et al., 2018; Villani et al., 2019; Wang and Bi, 2019; Zhang et al., 2019), and also supports recent studies that claimed abstract concepts should be studied using a category-specific approach (Ghio et al., 2016; Desai et al., 2018; Mkrtychian et al., 2019). In this sense, social experience is expected to be an important embodied dimension to characterize SA concepts and distinguish them from other different categories of abstract concepts.

However, the results of the two experiments consistently indicated that no significant differences in response times were observed between semantically related and unrelated negative picture-SA/EA words pairs, suggesting that the automatic spreading of activation did not occur between negative primes and targets. This is probably due to the negativity of the information encoded in the primes, which inhibits such spreading activation. As expected, the effect of social experience on abstract concepts was modulated by their valence, which is in line with previous findings with regard to the different semantic priming between positive and negative primes (Sass et al., 2012). For example, Rossell and Nobre (2004) analyzed the effect of valence on semantic priming and observed a significant priming effect for positive stimuli, a null effect for fearful stimuli, and an inhibited effect for sad stimuli. According to the spreading inhibition hypothesis (Clore and Storbeck, 2006), one possible reason for the null effects for negative picture-word pairs in Experiment 1 and 2 is that there exists a different organization of positive and negative information in semantic memory. Positive information of primes can increase the accessibility of prime-target pairs, by contrast, negative information of primes inhibits it, which makes the spreading of activation between connected nodes more difficult. Additionally, some researchers clearly proposed that people seem to perceive positive information as more compatible with negative information (Unkelbacha et al., 2020), because the human brain is more reactive to the valence of positive relative to negative words (Yang et al., 2013).

## Conclusion

In summary, our study demonstrated that social experience exerted a different role in the recognition of SA and EA concepts in a lexical priming-decision task. Specifically, the recognition of SA concepts could benefit from semantically related SS pictures in positive priming context, whereas EA concepts did not. This finding suggests that positive SA and EA concepts are grounded in different degrees of social experience. Thus, as a newly emerging embodied dimension, social experience may be capable of characterizing the key features of SA concepts, thus effectively distinguishing them from different kinds of abstract concepts.

Although it is increasingly apparent that social experience is a constitutive part of abstract concepts, work in this area is still in its early stages. Our study provides preliminary evidence to support the varied facilitating effect of social experience on different sub-categories of abstract concepts, and such effect is limited to positive SA concepts in semantic priming. One important limitation of the present study is that only a lexical decision-priming task and behavioral measure were employed, which may limit the application of our findings to the studies of the relationship between abstract concepts and sociality. Moreover, apart from social scene pictures, social situational sentences or social experience also provide specific social experiential information. Therefore, further research should extend this topic to other cognition tasks and materials, and further explore the role of social experience in the grounding of different kinds of abstract concepts.

## Data availability statement

The datasets presented in this study can be found in online repositories. The names of the repository/repositories and accession number(s) can be found in the article/Supplementary material.

## Ethics statement

The studies involving human participants were reviewed and approved by the Ethics Committee of Xi’an Jiaotong University. The patients/participants provided their written informed consent to participate in this study.

## Author contributions

ZY designed the study, analyzed the data, wrote the paper, and approved the final version and is accountable for all aspects of the work. FW were involved in data analysis work and corrected grammar. YC, PY, and RZ helped to collect and interpret the data. All authors contributed to the article and approved the submitted version.

## Conflict of interest

The authors declare that the research was conducted in the absence of any commercial or financial relationships that could be construed as a potential conflict of interest.

## Publisher’s note

All claims expressed in this article are solely those of the authors and do not necessarily represent those of their affiliated organizations, or those of the publisher, the editors and the reviewers. Any product that may be evaluated in this article, or claim that may be made by its manufacturer, is not guaranteed or endorsed by the publisher.
