# Supplementary material for: Effects of social experience on abstract concepts in semantic priming
Source: Front Psychol. 2022 Sep 2;13:912176. doi: 10.3389/fpsyg.2022.912176 (PMC9480607; doi:10.3389/fpsyg.2022.912176)
Supplement: Supplementary file 1 [file Data_Sheet_1.docx]

**Appendix A**

**Instructions for the picture naming task**

In this experiment, you will see about 100 pictures. Your task is to view each picture then provide 2 (minimum) – 5 (maximum) words you believe are related to the picture, and type them in the space below you. Please report words in the order you deem most appropriate.

Please complete it carefully in 3 days using self-paced. After submitting your answer, you will receive 50 RMB for your participation.

For example, if the picture includes two girls, do not report “girls”. Rather, we would like you to focus on words that refer to emotions one might be feeling in the scenario, or words that sum up the scenario as a whole. That is, please do not report objects, people, or physical actions depicted in the scene.

Here is an example picture with good responses.


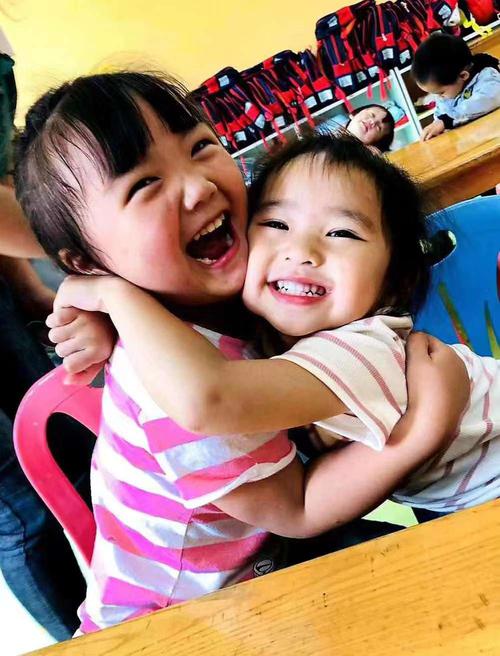


1. 友谊 friendship

2. 友善 amicable

3. 友好 kind

4. 好友 close friend

5. 情谊 friendly feelings

Notice how we did not include words like, “小女孩girl”, “教室classroom” or “拥抱hug”.

**Appendix B**

The instructions were translated into English on the basis of the original Chinese description:

The ratings of affective variable for pictures

In this experiment, you will see several pictures. Your task is to assess each picture’s valence and arousal using a 9-point scale (1 meant extremely negative/calm, 9 meant extremely positive/arousing). Valence is the extent to which the picture makes you feel negative/unpleasant (e.g., anguished, scared) or positive/pleasant (e.g., happy, pride), whereas arousal is the extent to which the picture makes you feel calm (relaxed, composed) or excited (stimulated, agitated).

Note, there are no right or wrong answers, you should make a quick assessment based on your first reaction upon seeing the word.

An example picture is as follows:


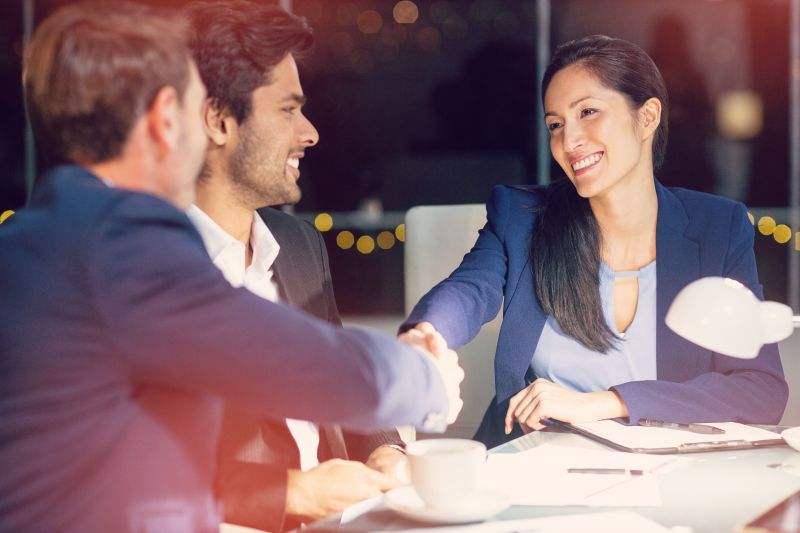
If you think that the picture has a very positive meaning, you may choose 8. If you feel some exciting when you see the picture, you may choose 7.

The ratings of affective and lexical variables for words

In this experiment, you will see several words. Your task is to assess each word’s valence, arousal, abstractness, familiarity, and referent using a 9-point scale (1 meant extremely negative/calm/abstract/unfamiliar/point to inner feelings, 9 meant extremely positive/arousing/concrete/familiar/derived from human interaction).

Valence is the extent to which the word makes you feel negative/unpleasant or positive/pleasant; Arousal is the extent to which the word makes you feel calm/relaxed or excited/stimulated; Abstractness is the extent to which the word is abstract/intangibles in the real world; Familiarity is the level of familiarity when you read the word; Referent is the extent to which the word points to inner feelings or derives from human interaction. Note, there are no right or wrong answers, you should make a quick assessment based on your first reaction upon seeing the word.

Two example words are as follows:

| Examples | Valence | Arousal | Abstractness | Familiarity | Referent |
| --- | --- | --- | --- | --- | --- |
| honor | 8 | 7 | 2 | 8 | 8 |
| sad | 2 | 7 | 3 | 7 | 2 |

**Appendix C**

Part of picture-word pairs used in the present experiments.

| Pairs’ type | Prime picture | Target word | English translation | Semantic relationship |
| --- | --- | --- | --- | --- |
| Positive SS-SA | 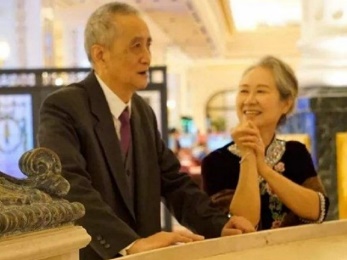 | 爱慕 | adore | 6.23 |
|  | 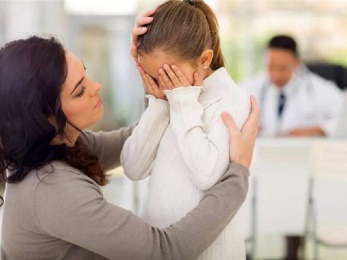 | 安慰 | comfort | 6.43 |
|  | 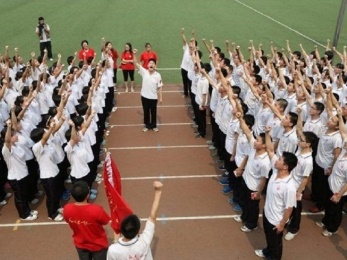 | 奋斗 | struggle | 6.21 |
|  | 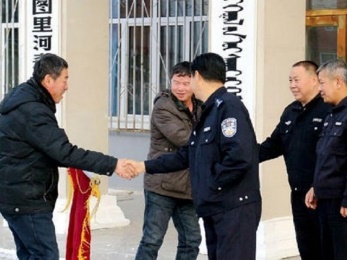 | 感激 | gratitude | 6.56 |
|  | 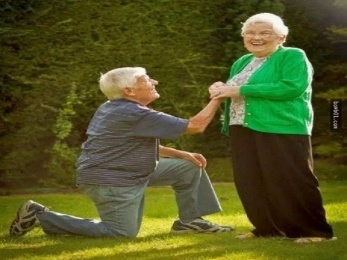 | 浪漫 | romance | 6.45 |
|  | 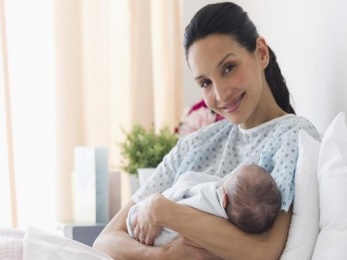 | 母爱 | maternal love | 6.22 |
|  | 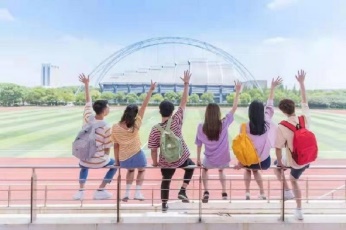 | 青春 | youth | 6.34 |
|  | 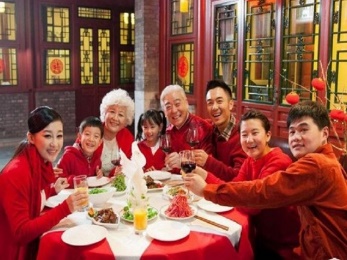 | 团聚 | reunite | 6.40 |
|  | 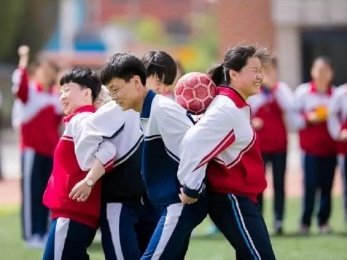 | 协作 | cooperation | 6.21 |
|  | 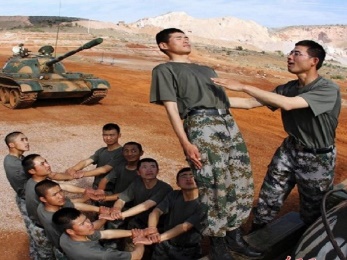 | 信任 | trust | 6.16 |
|  | 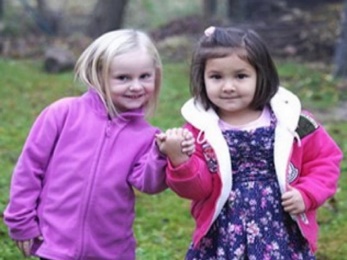 | 友谊 | friendship | 6.45 |
|  | 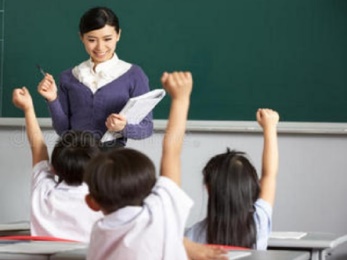 | 自信 | confident | 6.22 |
|  | 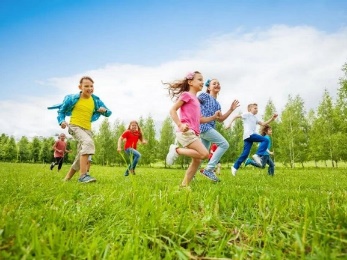 | 自由 | freedom | 6.49 |
| Negative SS-SA | 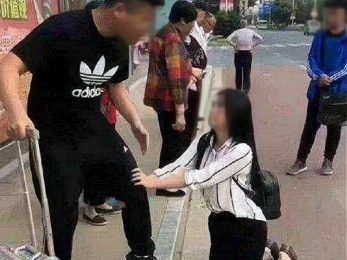 | 卑微 | humble | 6.23 |
|  | 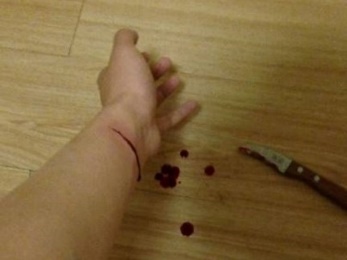 | 悲剧 | tragedy | 6.24 |
|  | 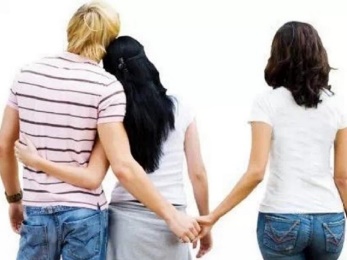 | 背叛 | betrayal | 6.19 |
|  | 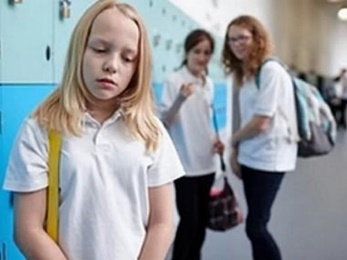 | 嘲讽 | taunt | 6.26 |
|  | 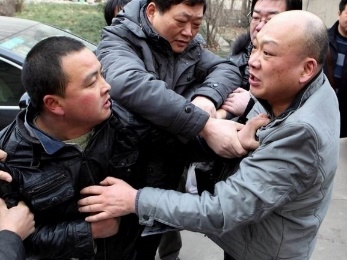 | 冲动 | impulse | 6.30 |
|  | 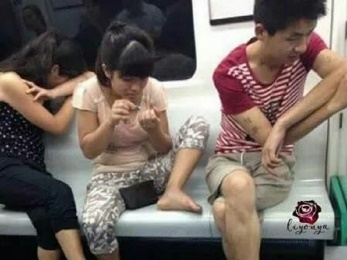 | 粗俗 | vulgar | 6.32 |
|  | 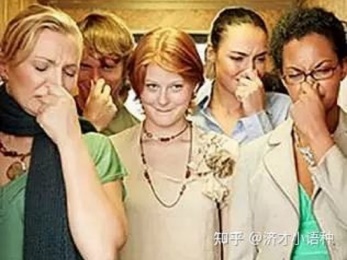 | 尴尬 | embarrassed | 6.28 |
|  | 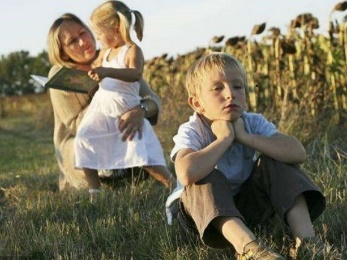 | 忽视 | neglect | 6.24 |
|  | 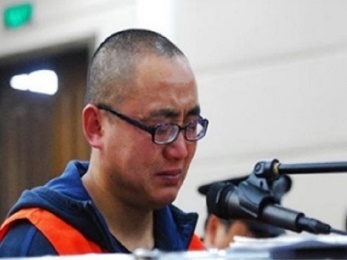 | 悔恨 | regret | 6.14 |
|  | 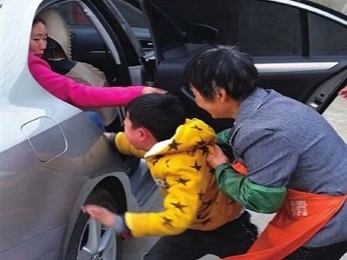 | 离别 | separation | 6.29 |
|  | 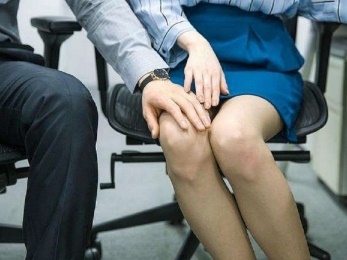 | 侵犯 | harass | 6.16 |
|  | 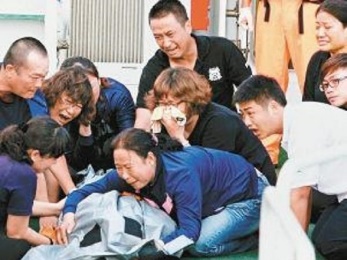 | 丧失 | bereaved | 6.30 |
|  | 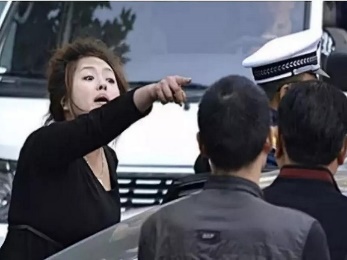 | 嚣张 | arrogant | 6.21 |
|  | 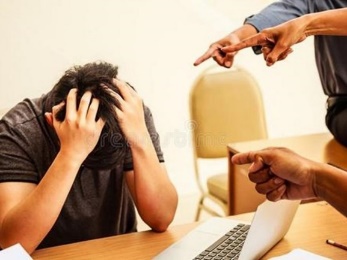 | 指责 | accuse | 6.26 |
| Positive EE-EA | 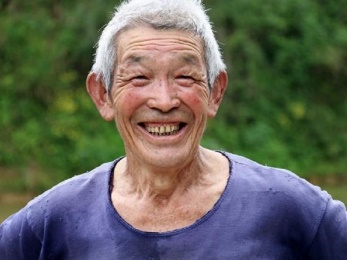 | 高兴 | glad | 6.43 |
|  | 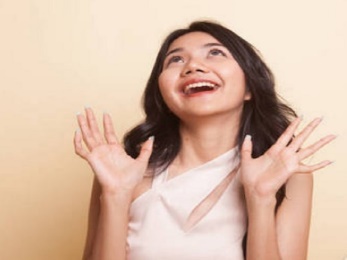 | 欢喜 | joyful | 6.44 |
|  | 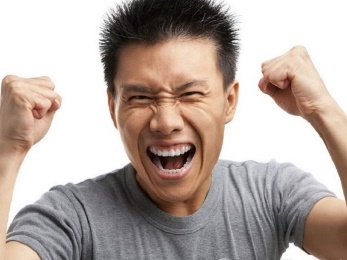 | 激动 | fevered | 6.39 |
|  | 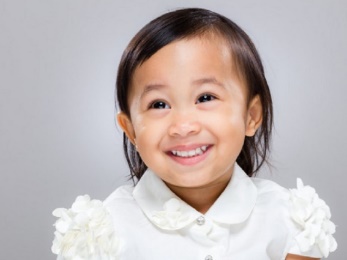 | 开心 | delighted | 6.26 |
|  | 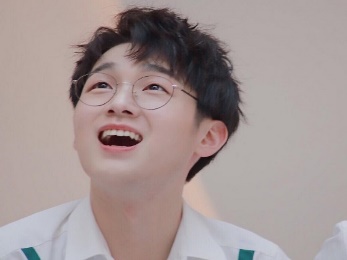 | 渴望 | desire | 6.20 |
|  | 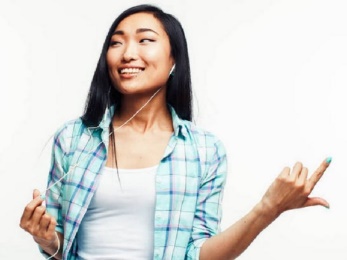 | 快活 | cheerful | 6.22 |
|  | 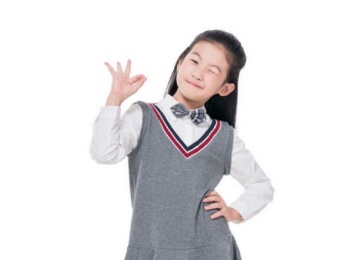 | 快乐 | happy | 6.28 |
|  | 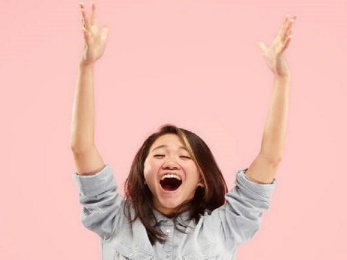 | 狂喜 | ecstasy | 6.53 |
|  | 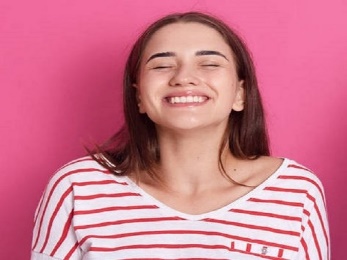 | 满足 | content | 6.44 |
|  | 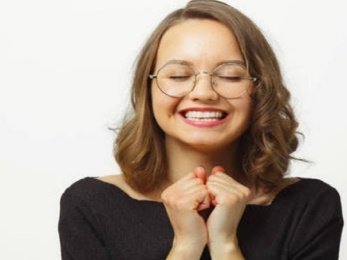 | 期待 | expect | 6.39 |
|  | 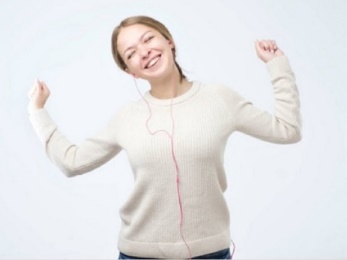 | 惬意 | cozy | 6.36 |
|  | 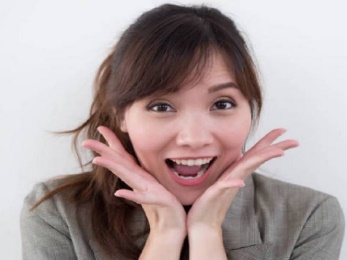 | 喜悦 | delightful | 6.40 |
|  | 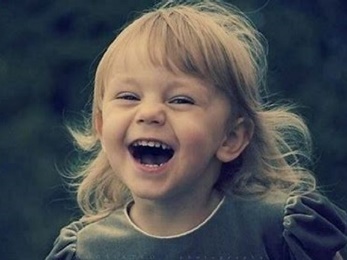 | 愉快 | jolly | 6.32 |
|  | 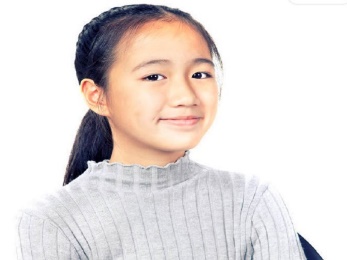 | 愉悦 | pleasant | 6.28 |
| Negative EE-EA | 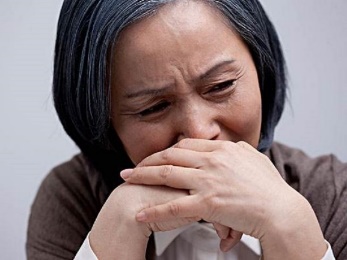 | 哀伤 | grief | 6.23 |
|  | 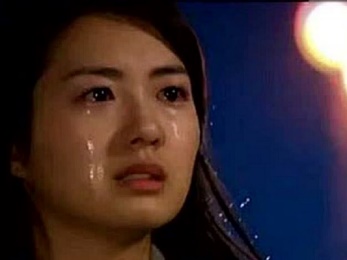 | 悲伤 | heartbroken | 6.24 |
|  | 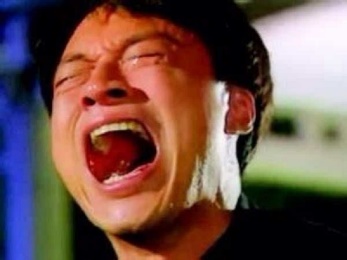 | 悲痛 | sorrowful | 6.19 |
|  | 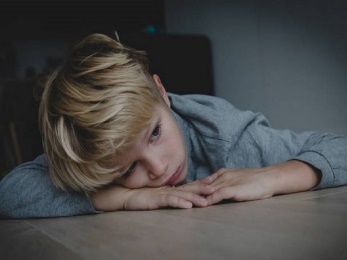 | 烦闷 | annoyed | 6.16 |
|  | 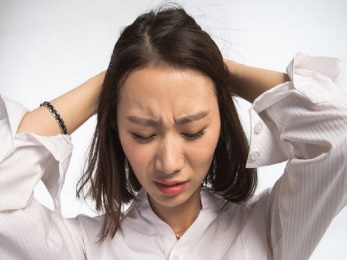 | 烦躁 | restless | 6.20 |
|  | 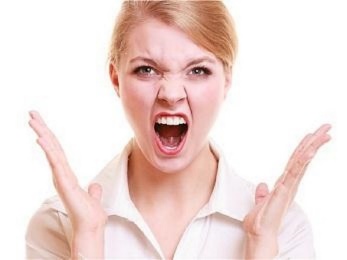 | 愤怒 | resentful | 6.32 |
|  | 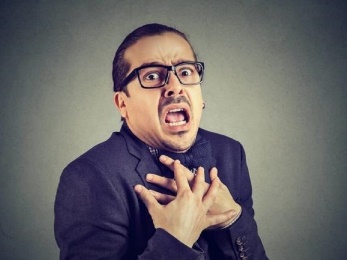 | 惊恐 | scared | 6.28 |
|  | 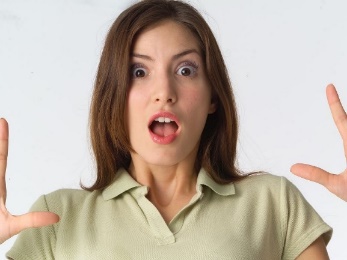 | 惊讶 | amazed | 6.23 |
|  | 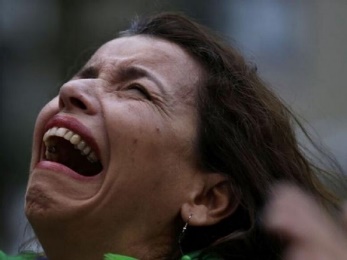 | 绝望 | despaired | 6.24 |
|  | 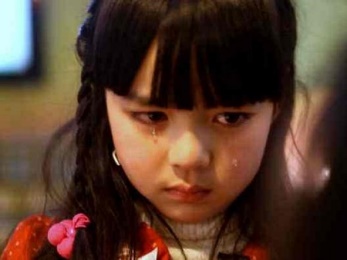 | 难过 | sorry | 6.19 |
|  | 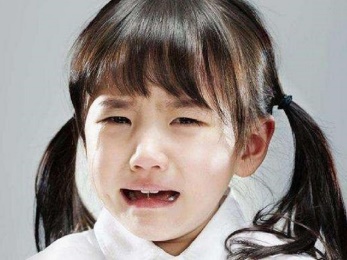 | 伤心 | sad | 6.26 |
|  | 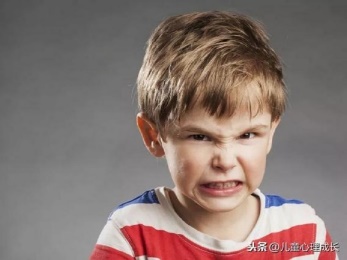 | 生气 | angry | 6.40 |
|  | 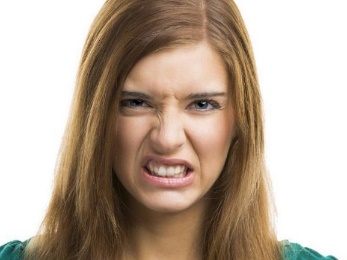 | 厌恶 | disgusted | 6.22 |
|  | 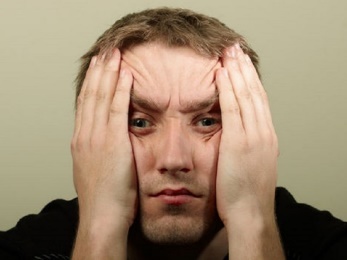 | 忧虑 | worried | 6.18 |
